# Additional fundophrenicopexia, after Nissen fundoplication, reduces postoperative dysphagia and re-operation rate in the long-term follow up

**DOI:** 10.1007/s00464-021-08598-5

**Published:** 2021-06-22

**Authors:** Milena Nikolic, Aleksa Matic, Ivan Kristo, Matthias Paireder, Reza Asari, Bogdan Osmokrovic, Georg Semmler, Sebastian F. Schoppmann

**Affiliations:** Department of Surgery, Division of General Surgery, Medical University of Vienna, Waehringer Guertel 18-20, 1090 Vienna, Austria

**Keywords:** GERD, Nissen fundoplication, Division of the short gastric vessels, Fundophrenicopexia, Dysphagia, Re-herniation

## Abstract

**Background:**

Various technical modifications of Nissen fundoplication (NF) that aim to improve patients’ outcomes have been discussed. This study aims to evaluate the effect of division of the short gastric vessels (SGV) and the addition of a standardized fundophrenicopexia on the postoperative outcome after NF.

**Methods:**

283 consecutive patients with GERD treated with NF were divided into four groups following consecutive time periods: with division of the SGV and without fundophrenicopexia (group A), with division of the SGV and with fundophrenicopexia (group B), without division of the SGV and with fundophrenicopexia (group C) and without division of the SGV and without fundophrenicopexia (group D). Postoperative contrast swallow, dysphagia scoring, GEDR-HRQL and proton pump inhibitor intake were evaluated. A comparative analysis of patients with division of the SGV and those without (161 A + B vs. 122 C + D), and patients with fundophrenicopexia and those without (78 A vs. 83 B and 49 C vs. 73 D) was performed.

**Results:**

Fundophrenicopexia reduced postoperative dysphagia rates (0 group C vs. 5 group D, *p* = 0.021) in patients where the SGV were preserved and reoperation rates (1 group B vs. 7 group A, *p* = 0.017) in patients where the SGV were divided. There was no significant difference in the postoperative rates of heartburn relief, dysphagia, gas bloating syndrome, interventions, re-fundoplication and the GERD-HRQL score between groups A + B and C + D, respectively.

**Conclusion:**

Standardized additional fundophrenicopexia in patients undergoing Nissen fundoplication significantly reduces postoperative dysphagia in patients without division of the SGV and reoperation rates in patients with division of the SGV. Division of the SGV has no influence on the postoperative outcome of NF.

Gastroesophageal reflux disease (GERD) represents a public health issue with an increasing prevalence and high economic burden. [1–4] Patients have choose between medical and surgical therapy in an effort to improve their quality of life and prevent possible complications of long-term acid exposure as well as anti-acid treatment. [5] However, less than 1% of GERD patients ultimately opt for surgical GERD treatment. [2, 3, 6] Although the reason behind the decrease in anti-reflux operations noted over the last decades seems multifactorial, one possible explanation is the fear of short term adverse effects such as re-herniation and need for reoperation as well as long-term side effects like dysphagia and gas-bloat syndrome. [7–10]

The Nissen fundoplication (NF) underwent countless alterations in an attempt to reduce side effects, increase effectiveness and subsequently minimize the therapy gap between medical and surgical GERD treatment. [11–13] The original surgical technique consisted of the division of the phrenoesophageal ligament, mobilization of the esophagus, without division of the short gastric vessels (SGV) and wrapping of the anterior and posterior wall of the stomach 360° around the lower 6 cm of the esophagus using 4 or 5 interrupted sutures. [11] As dysphagia rates were high following such a procedure, partial 270° Toupet fundoplication and 120° Dor fundoplication were developed in Europe. [11, 13] Furthermore, DeMeester and Johnson modified the procedure to a loose “floppy Nissen” by dividing the SGV and proving that a maximal wrap of 2 cm was enough to provide reflux control but prevent dysphagia and gas bloating. [14, 15]

Nevertheless, the division of the SGV remains controversial and surgeon-dependent. [16–18] Although it has been claimed that dividing the SGV may minimize the risk of dysphagia [19], different studies failed to demonstrate both short and long-term benefits for patients that underwent this maneuver. [17, 18, 20–22] Moreover, some trials have reported a higher incidence of abdominal bloating and recurrent hiatal hernia as well as increased operating time including division of the SGV. [23–25] Furthermore, added posterior gastropexy aimed to reduce the re-herniation rate and lengthen the intraabdominal esophagus. Without worsening the dysphagia rates, posterior gastropexy has shown to have a positive impact on the postoperative outcome of NF. [26–28] Encouraged by these results we integrated suturing the fundoplication wrap with the right crus (fundophrenicopexia) with two non-resorbable sutures.

The aim of this study was to analyze the differences in postoperative dysphagia rates, reflux control, re-fundoplication rate as well as the degree of overall satisfaction in GERD patients who underwent laparoscopic Nissen fundoplication (NF) with and without division of the short gastric vessels and/or fundophrenicopexia in a high volume specialized reflux center.

## Methods

### Patient selection

**Fig. 1:**
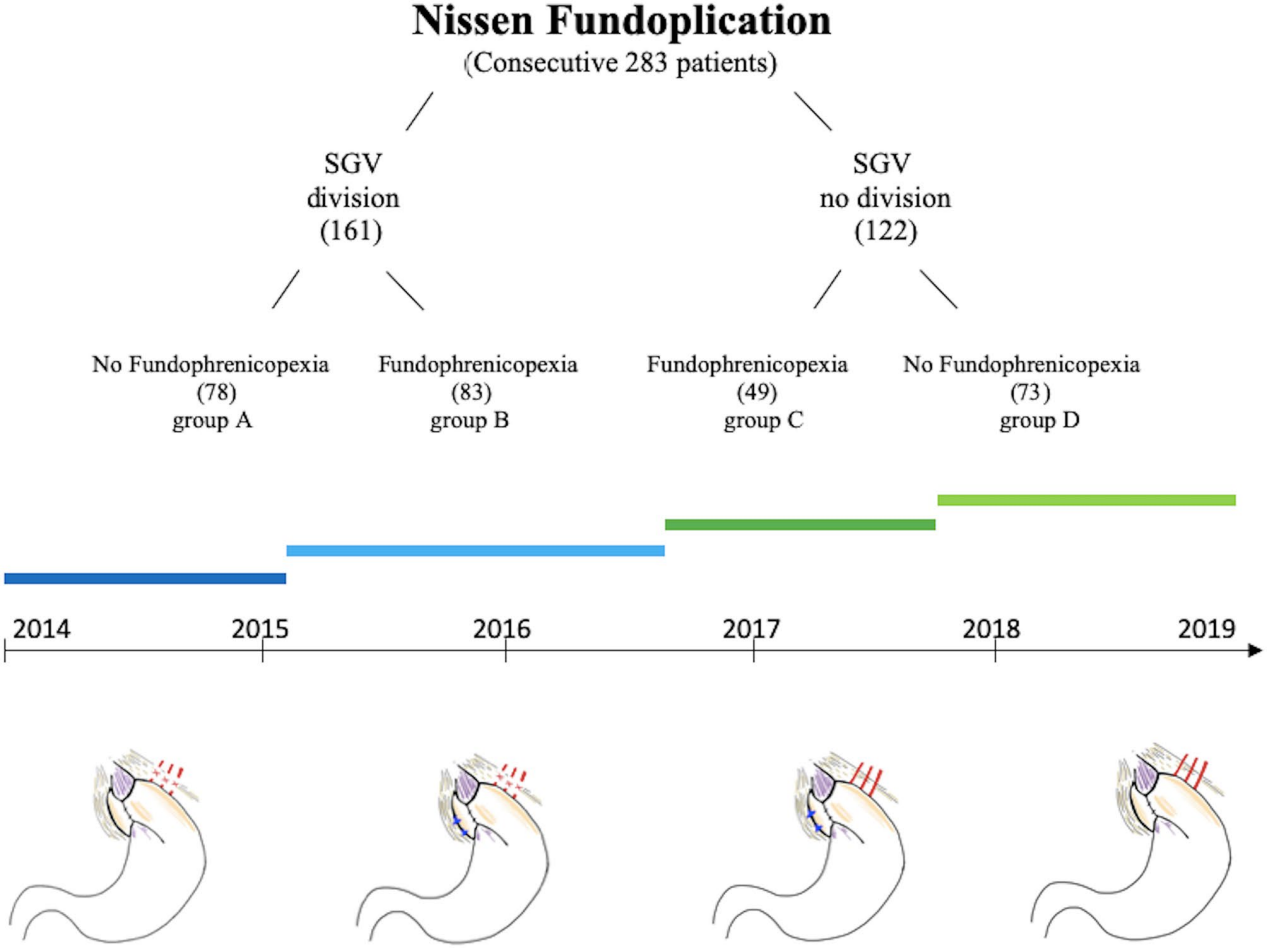
Patient inclusion chart including fundoplication modifications

All consecutive patients that underwent NF between the years 2014 and 2019 were included. During this time period the procedure was consequently modified four times (see patients inclusion chart Fig. 1). From 01/2014 until 02/2015 all NF included full mobilization of the fundus with division of the upper half of the SGV. From 02/2015 until 08/2016 all NF included division of the SGV as well as fundophrenicopexia, with two non-absorbable sutures between the fundoplication wrap and right crus. From 08/2016 until 09/2017 we ceased fully mobilizing the fundus by dividing the SGV, while fundophrenicopexia was still performed. Finally from 09/2017 until 2019 no division of the SGV and no fundophrenicopexia was performed (see illustration of the four fundoplication modifications Fig. 1).

### Preoperative assessment

All patients received a standardized interview, clinical examination, an upper GI endoscopy, a video esophagogram and esophageal functioning testing consistent of a manometry and a 24-h-impedance-pH-metry. GERD was diagnosed by positive pH results or increased total reflux episodes with positive symptom correlation on esophageal functioning tests, presence of esophagitis on endoscopy or typical GERD symptoms sensitive to PPI medication. Hiatal hernia was diagnosed with high precision using both upper GI endoscopy and high-resolution manometry.

### Surgery

All procedures were performed by one of the senior surgeons who were part of the specialized upper GI surgical unit. (SFS, RA). The surgical approach was laparoscopic in all cases. All procedures were standardized regarding the surgeon’s and patient’s positions (anti-Trendelenburg), trocar sites and used instruments. These procedures were accomplished by hiatal dissection and crural closure with 1–5 stitches using non-absorbable sutures. All cases were performed without the use of an esophageal bougie.

### Nissen fundoplication

LNF was performed in a highly standardized technique as described recently. [29] In brief: both crura of the diaphragm were dissected using the ultrasonic dissector to expose the distal esophagus. Special care was taken to achieve an adequate “intraabdominalisation” of the lower esophagus of at least 3 cm in length. An extra-short wrap, measuring 1.5 cm in a maximum with the naked eye was created using 2 close stiches with non-absorbable sutures. The first stich included the anterior esophageal wall. The vagal nerve was always identified and included in the wrap. After the operation was completed, a blunt laparoscopic instrument was placed between the posterior esophageal wall and the wrap to determine the looseness of the fundoplication.

### Postoperative care

All patients undergoing LNF received a restricted semiliquid food diet for the first 10 days, slowly progressing to solid food to avoid dysphagia during the development of mucosal edema. On the first postoperative day a contrast swallow with diatrizoate was performed in all patients. After at least one overnight stay, patients were discharged from the hospital once they displayed an unremarkable postoperative contrast swallow.

### Postoperative assessment

For analysis, all patients were divided into four groups following the consecutive time periods (see patients chart): NF with division of the SGV and without fundophrenicopexia (group A), NF with division of the SGV and with fundophrenicopexia (group B), NF without division of the SGV, but with fundophrenicopexia (group C) and NF without division of the SGV and without fundophrenicopexia (group D). These groups were included in a comparative analyses (A + B vs. C + D). Furthermore, we performed a comparative analysis of patients that underwent NF with fundophrenicopexia and those without, within the two main groups (A vs. B and C vs. D).

The median follow-up time was 5 years (IQR, 1.8–10). Follow-up was performed by the same physician using a standardized interview assessing postoperative gastrointestinal symptoms, proton pump inhibitor intake (PPI), GERD-Health-related-Quality-of-Life (GERD-HRQL) and overall Alimentary Satisfaction (AS). The overall AS was assessed using a scale from 0 – 10 of Greene et al., where the score 0 indicated an intolerable alimentary function while a score of 10 indicated complete satisfaction. [30] The frequency and severity of postoperative dysphagia was assessed using the classification of Saeed et al., where the ability to swallow can be scored from 0 to V, where 0 is inability to swallow and V is normal swallowing (Table 4.). [31]

Adverse events such as complications, hospital readmission, emergency operation or elective reoperation were documented. Patients with recurrent symptoms received upper GI endoscopy as well as esophageal functioning tests.

### Statistical analysis

Statistical analysis was performed using SPSS® statistics 20.0 (IBM, Armonk, NY). Data were described using median (interquartile range (IQR)) or mean (range). Statistical analysis appropriate for non-parametric data were used. Categorical variables were assessed using the Fisher exact test and continuous data using the Wilcoxon Rank test as appropriate. Statistical significance was defined as a *p*-value < 0.05.

## Results

A total of two hundred and eighty three (*n* = 283) consecutive patients that underwent laparoscopic NF were included for comparative analyses. One hundred and sixty one (57% groups A + B) patients underwent division of the short gastric vessels whereas in one hundred and twenty-one (43% groups C + D) patients no division of the short gastric vessels had been performed. There was no significant difference in age, sex, body mass index (BMI), preoperative total number of reflux episodes, hiatal hernia size and presence of Barrett’s esophagus between the groups. Each group was then further divided into a subgroup depending if a fundophrenicopexia was performed. Demographics and preoperative findings are shown in Table 1.

**Table 1:** Demographic data and results of preoperative diagnostics of all patients

	Division of SGV	No division of SGV	
Total *n* = 283 (100%)	*N* = 161 (57%)	*N* = 122 (43%)	
Fundophrenicopexia	83	49	
Sex (M vs. F)	95 vs. 66	74 vs. 48	*p* = 0.299
Median Age (IQR)	52	55	*p* = 0.099
Median BMI (IQR)	26.6	25.9	*p* = 0.162
Median size of HH(cm)	3	3	*p* = 0.444
Median total pH < 4%	7%	11%	*p* = 0.002
Median total Reflux episodes	71	70	*p* = 0.929
Presence of BE	27	21	*p* = 0.639
Use of PPIs	136	115	*p* = 0.535
Most common symptoms			
Heartburn	158	109	
Regurgitations	122	61	
Respiratory symptoms	62	21	
Dysphagia	18	9	

### Operative parameters

The surgical approach was laparoscopic in all patients. The median operation time (OR) time was 55 min (IQR, 40 – 80 min). There was a significant difference in the operation time when comparing the two groups (55 min groups A + B vs. 40 min groups C + D, *p* = 0.002). No intraoperative complications were seen. Postoperative swallow X-ray showed regular postoperative results in all patients.

### Symptom relief

The median follow-up time was 5 years (IQR, 1.8–10). Heartburn was fully eliminated in two hundred and twenty-nine patients (*n* = 229/277, 83%), while regurgitations were improved in one hundred and sixty-nine patients (*n* = 169/183, 92%) and fully eliminated in one hundred and forty-three (*n* = 143/183, 78%) of the patients. When analyzing the pre- and postoperative symptoms we found a statistically significant difference between all three symptoms. A comparison of the two most reported symptoms before and after NF is shown in Table 2.

**Table 2:** Comparison of preoperative and postoperative symptoms (*n* = 283)

	Division of SGV			No division of SGV		
	Preoperative	Postoperative		Preoperative	Postoperative	
Heartburn	158	22	*p* = 0.0001	109	18	*p* = 0.0001
Regurgitations	122	33	*p* = 0.0001	61	21	*p* = 0.0001
Respiratory symptoms	62	30	*p* = 0.0001	21	10	*p* = 0.0001

### Side effects

Forty (14%) patients reported they were unable to belch/vomit and thirty-five (12%) patients complained about increased daily gas bloating. There was no difference in the postoperative inability to belch/vomit (17 groups A + B vs. 23 groups C + D, *p* = 0.071) or in daily gas bloating (19 groups A + B vs. 16 groups C + D, *p* = 0.740) between the groups. Summary of postoperative side effects are presented in Table 3.

**Table 3:** Postoperative side-effect rate at median follow-up of 5 years

	Division of SGV	No division of SGV	
Persistent dysphagia	6	5	*p* = 0.873
Gas-bloat syndrome	19	16	*p* = 0.740
Ability to belch/vomit	17	23	*p* = 0.071
Intervention	3	3	*p* = 0.567
Re-fundoplication	8	8	*p* = 0.236

### Dysphagia

Persistent dysphagia, defined as 0, I or II in the classification of Saeed et al., was reported in eleven (*n* = 11, 4%) patients at the time of follow-up. [31] The frequency and degree of postoperative dysphagia is shown in Table 4. When comparing the patients that had no division of the SGV we found a significant difference in the postoperative persistent dysphagia rate between group C (0%) and D (7%) in favor of group C (*p* = 0.021). There was no difference in the occurrence of persistent dysphagia when comparing patients with and without the division of the SGV (6 groups A + B vs. 5 groups C + D, *p* = 0.873). Furthermore, no difference in the persistent dysphagia rate was observed between group A (4%) and group B (4%) when comparing patients with division of the SGV (*p* = 0.938).

**Table 4:** Frequency and degree of postoperative dysphagia based on the classification of Saeed et al.

0 = Unable to swallow	*n* = 0
I = Swallowing liquids with difficulty, solids impossible	*n* = 4
II = Swallowing liquids without difficulty, solids impossible	*n* = 7
III = Occasionally difficulty swallowing with solids	*n* = 22
IV = Rarely difficulty swallowing with solids	*n* = 66
V = Swallowing normally	*n* = 184

Out of 11 patients with persistent dysphagia, we observed two patients with slipping of the fundoplication wrap, only one of which underwent a surgical revision. One patient developed a rupture of the wrap 2 years after the primary operation with reflux reoccurrence and underwent a surgical revision of the wrap, after which he developed dysphagia and underwent a third operation 6 months after the revision, where a couple adhesions were divided. Two out of three patients with persistent dysphagia showing no morphological abnormalities in postoperative investigations underwent one or more endoscopic balloon dilatations, one of which underwent a conversion to a Toupet fundoplication two years later. Five patients with persistent dysphagia did not undergo further diagnostic evaluation.

No correlation was found between preoperative ineffective esophageal motility (IEM) and postoperative dysphagia (*p* = 0.645), as only 1 out of 11 patients with persistent dysphagia was diagnosed with IEM prior to the operation.

### Interventions and revision operation

Endoscopic dilatation was performed in six patients (2%) with persistent dysphagia, with four of them having a successful outcome, leaving two patients with persistent dysphagia at the time of follow-up. Sixteen (6%) patients required re-fundoplication operation. Six patients underwent revision operation due to dysphagia, four patients developed re-herniation of the fundus/wrap, four patients developed rupture of the fundoplication wrap and reoccurrence of reflux symptoms, one patient developed slipping of the fundoplication wrap and lastly, one patient developed gastric carcinoma and needed gastric resection. Nine patients (*n* = 9/16, 56%) underwent revision operation within one year after the primary operation, five patients (*n* = 5/16, 31%) underwent revision operation one to three years after the primary operation and two patients (*n* = 2/16, 13%) underwent revision operation three to five years after the primary operation.

There was no difference in the postoperative rate of endoscopic dilatation between the two groups (3 groups A + B vs. 3 groups C + D, *p* = 0.567), or between either of the subgroups. We found no difference in the rates of reoperation between the two groups (8 groups A + B vs. 8 groups C + D, *p* = 0.236). Even though there was a clear trend towards a reduced revision rate in patients without division of the SGV and fundophrenicopexia when compared to those without division of the SGV and without fundophrenicopexia (1 group C vs. 7 group D, *p* = 0.075), it missed statistical significance. However, in the group of patients that had undergone division of the SGV and fundophrenicopexia a significant reduced reoperation rate when compared to those with division and without fundophrenicopexia was found (1 group B vs. 7 group A, *p* = 0.017).

### Quality of life

Prior to the operation 98 patients had completed the GERD-HRQL score. The preoperative median total GERD-HRQL score was 19.5 (IQR 13 – 25). Laparoscopic NF led to a significant reduction of the GERD-HRQL total score (19.5 vs. 2, *p* = 0.00). When comparing patients with and without division of the SGV we see no difference in the postoperative GERD-HRQL score (2 groups A + B vs. 2 groups C + D, *p* = 0.802). Moreover, the median AS was rated 9, also with no difference between the groups (9 groups A + B vs. 10 groups C + D, *p* = 0.074). Two hundred and fifty-six patients (*n* = 256, 90%) reported being satisfied with their outcome after NF, with minimal difference between the groups (141 groups A + B vs. 115 groups C + D, *p* = 0.049). Two hundred and thirty-nine (*n* = 239, 84%) claimed they would undergo the same operation again, with no difference between the groups (130 groups A + B vs. 109 groups C + D, *p* = 0.097). This proves a substantial increase in quality of life in our patients. One hundred and ninety-eight (*n* = 198/251, 79%) patients reported to be completely free of PPIs postoperatively, while twenty-three (*n* = 23/251, 9%) patients needed regular PPI use. No difference was observed in the postoperative rate of PPI use between the two groups (31 groups A + B vs. 22 groups C + D, *p* = 0.784).

## Discussion

The laparoscopic NF has been the gold standard in anti-reflux surgery to date, achieving up to 20 years of effective reflux control and significant improvement of quality of life. [5, 21, 32–34] Nonetheless, the NF has also been associated with certain adverse effects, such as persistent dysphagia and gas-bloat syndrome. [7–10, 35] Furthermore, although minimal, a risk of fundoplication failure, defined as re-herniation, slipping of the wrap or reoccurrence of symptoms requiring reoperation also exists. [8, 36–38] Throughout history surgeons have been trying to optimize the procedure, modifying certain segments to reduce the complication rate. [11–13] Still no consensus exists whether the SGV should be ligated or not or whether the fundoplication wrap or/and remaining stomach should be fixated to the crus or not. [21, 26–28, 39, 40] Thus, it ultimately depends on the surgeon how the NF is performed precisely. Creating a 360° tension-free wrap, no longer then 1,5 cm length, with two non-absorbable sutures, including the esophagus in the first one remained the basis of our NF from beginning to date. Similarly though, the procedure underwent modifications throughout the years: (1) full mobilization of the fundus with division of the short gastric vessels, but without fundophrenicopexia, (2) full mobilization of the fundus with division of the short gastric vessels and fundophrenicopexia, (3) mobilization of the fundus without diving the short gastric vessels, but with fundophrenicopexia and finally (4) mobilization of the fundus without diving the short gastric and fundophrenicopexia. Therefore, in this study we aim to compare the postoperative outcome of patients that underwent these four modifications of the NF in our high output surgical reflux center.

In our study we observed a total of eleven patients (4%) with postoperative persistent dysphagia at the time of follow-up. These results coincide with previous literature where dysphagia after NF ranges from 2 to 11%. [29, 34, 41] The hypothesis that division of the SGV is needed to achieve a tension-free fundoplication wrap and thus minimize the risk of postoperative dysphagia led to many surgeons integrating this step in the NF. [14, 15, 19] However, multiple randomized prospective studies have shown no impact of this intraoperative modification in the reduction of the above mentioned adverse effect. [16–18, 22, 39, 40] Moreover, a higher incidence of postoperative bloating, re-herniation and longer operating time has been described in patients that underwent the additional modification of the NF. [20, 23–25] Our results are similar to previous literature showing no difference in postoperative dysphagia rates in patients with and those without division of the SGV (6 groups A + B vs. 5 groups B + C, *p* = 0.873). Similarly, we also found longer operating time in patients where the SGV were divided (55 min groups A + B vs. 40 min groups C + D, *p* = 0.002). However, in contrast to some studies we found no significant difference in postoperative gas bloating (19 groups A + B vs. 16 groups C + D, *p* = 0.740) or reoperation between the two groups (8 groups A + B vs. 8 groups C + D, *p* = 0.567). Additionally, no difference was seen in the postoperative inability to belch/vomit (17 groups A + B vs. 23 groups C + D, *p* = 0.071). These results show us that additional division of the SGV has no benefit on the postoperative side-effect rate after NF.

Intrathoracic wrap migration/herniation, wrap disruption and telescoping/slipping have been shown as the most common causes of fundoplication failure. [42] As the gastroesophageal junction is thoroughly dissected and the esophagus maximally intraabdominalized, the positive intraabdominal pressure can cause the free wrap to migrate to the negative pressure, into the thorax. [37] In order to minimize this risk we added extra one or two sutures between the fundoplication wrap and the right crus – fundophrenicopexia in our NF. In our series we had sixteen patients (6%) that needed reoperation and six (2%) needing balloon dilatation. These results are also similar to previous literature showing revision rates from 3 – 6%, or in long-term follow-up from 5 – 15%. [43, 44] Interestingly, when looking closer at our group with division of the SGV and comparing the two subgroups with patients undergoing additional fundophrenicopexia and those without, we found a significant difference in the revision rate between the patients (1 group B vs. 7 group A, *p* = 0.017). The most common cause for reoperation were fundoplication wrap disorders: re-herniation, slipping and disruption (*n* = 4/7, 60%). This finding shows us that additional fundophrenicopexia in patients where full fundus mobilization is preformed anchors the fundoplication in its intraabdominal place reducing the risk of migration or slipping. Another interesting finding was the difference in persistent dysphagia rates in patients where no division of the SGV was performed (0 group C vs. 5 group D, *p* = 0.021). A possible explanation for this outcome could be that anchoring the wrap to the right crus relives the pressure from the spleen to the gastric fundus and prevents the kinking of the esophagus, when the SGV are not dissected, reducing further dysphagia. Nonetheless further prospective studies are needed to confirm the benefit of additional fundophrenicopexia in the NF.

We also found no correlation between preoperative IEM and postoperative dysphagia (*p* = 0.645). These findings confirm multiple previous studies showing no influence of the type of fundoplication on the outcome in patients with IEM [45–50], although still controversial.

In this study all the procedures were performed without the use of a bougie. Even though the results of a small prospective randomized study had shown lower dysphagia rates when applying a bougie during laparoscopic Nissen fundoplication, certain limitations of this study such as additional concurrent laparoscopic procedures that 34 patients underwent (unknown in which group), as well as the validity of the scoring system used to assess the dysphagia should be taken into consideration. [51] Furthermore, various studies reported no benefit of using a bougie in postoperative dysphagia rates as well as a higher risk of esophageal perforation potentially opposing the benefit. [52–56]

When observing the quality of life in our patients, we found that it significantly increases after NF (GERD-HRQL total score 19.5 vs. 2, *p* = 0.00). Moreover, the median AS was rated 9, two hundred and fifty-six patients ( 90%) reported being satisfied with their outcome after NF and two hundred and thirty-nine (84%) claimed they would undergo the same operation again. Finally when comparing the three most common GI symptoms – heartburn, regurgitation and respiratory symptoms, before and after FN we found a significant reduction of all three symptoms after the operation (with and without the division of the SGV (Table 2). These results further show that NF makes a substantial difference in symptom relief and GI quality of life in GERD patients.

A few limitations of our study, like its retrospective nature need to be taken into consideration. The various modifications were implemented over a time period and patients were not prospectively randomized, with the hopes of improving the outcome while reducing the side-effect rate. The patients in this study were selected consecutively and were prone to selection bias. Further prospective, controlled studies are needed to confirm the proposed benefits of such a modification in the NF with a higher level of evidence. Also, we relied purely on subjective postoperative patient evaluation of outcomes, as the majority of the patients were asymptomatic and invasive objective (EFTs) testing was difficult in the large cohort.

## Conclusion

At a median follow-up of five years the NF is shown to be a safe and effective operation in reflux control. We found additional division of the SGV has no benefit on the postoperative outcome of the NF. On the contrary additional suturing of the fundoplication wrap to the right crus reduces the risk of re-fundoplication when the SGV are ligated and reduces the risk of postoperative dysphagia when the SGV are not ligated. These results have established Nissen fundoplication performed without division of the SGV and with a standard fundophrenicopexia as the standard modification at our center.
